# Historic DNA for taxonomy and conservation: A case-study of a century-old Hawaiian hawkmoth type (Lepidoptera: Sphingidae)

**DOI:** 10.1371/journal.pone.0173255

**Published:** 2017-03-08

**Authors:** Anna K. Hundsdoerfer, Ian J. Kitching

**Affiliations:** 1 Senckenberg Natural History Collections Dresden, Dresden, Germany; 2 Department of Life Sciences, Natural History Museum, London, United Kingdom; University of Arkansas, UNITED STATES

## Abstract

Analysing historic DNA from museum specimens offers the unique opportunity to study the molecular systematics and phylogenetics of rare and possibly extinct taxa. In the Hawaiian fauna, the hawkmoth, *Hyles calida calida*, occurs on several of the main islands and is quite frequent, whereas *Hyles c*. *hawaiiensis* is restricted to the Island of Hawaii where it appears to be very rare. Analysis of mitochondrial DNA sequences shows that *Hyles c*. *hawaiiensis* differs from the nominotypical subspecies by an average p-distance of 2.8%, which is of a similar order of magnitude to that found between other species of *Hyles*, suggesting that *Hyles c*. *hawaiiensis* should perhaps be awarded species status, although more data are required for a formal taxonomic revision. Given the rarity of this taxon, these analyses should be undertaken urgently so that conservation measures can be implemented before it becomes extinct.

## 1. Introduction

Analysing historic DNA from museum specimens offers the unique opportunity to study molecular systematics and phylogenetics of rare and possibly extinct taxa. Controversial taxonomy can often be clarified by examining the historic DNA of type specimens, e.g. [1, 2]. The hawkmoth genus *Hyles* Hübner, 1819 (Lepidoptera: Sphingidae) has a global distribution with representatives on all continents (except Antarctica), as well as on many islands groups (e.g., Macaronesia, the Mascarenes, Hawaii). The genus currently comprises 32 species [3], which are generally identified using characters from the pattern and colour of the wings and abdomen. However, the taxonomy based on these morphological features is highly contentious, and there is considerable disagreement over both the number of species and how they are related (e.g., see overview in Hundsdoerfer et al. [1]). However, recent studies using DNA sequence data [4–6] have begun to unravel and clarify the complex phylogenetics of these moths, in which hybridization and introgression appear to play a considerable role.

Several species of *Hyles* are endemic to island groups, e.g. *Hyles biguttata* (Walker, 1856) on the Mascarenes and *H*. *livornicoides* (Lucas, 1892) in Australia, but it is only on the Hawaiian Islands that endemic *Hyles* have speciated to produce a radiation. The first to be described was *Deilephila calida* Butler, 1881, based on a single (male) specimen collected by the Reverend Thomas Blackburn from “the Hawaiian Islands” (Table 1). Next was *Deilephila wilsoni* Rothschild, 1894, which was also described from a single specimen (this time a female) from “Hawaii, Sandwich Islands” from Scott. B. Wilson. This species was redescribed shortly afterwards by Meyrick [7] as *Deilephila pyrias* Meyrick, 1899, based on “11 specimens; 9 Olaa, Hawaii, at 2000 feet; 2 Hilo, Hawaii, at 2000 feet; in January, June, September, November, and December”. Seven of these syntypes have been found in the collection of the Natural History Museum, London (NHMUK), as have the holotypes of *D*. *calida* and *D*. *wilsoni*, but the whereabouts of the remaining four syntypes of *D*. *pyrias* is unknown. Of the seven syntypes of *D*. *pyrias*, one from Hilo (NHMUK specimen registration no. BMNH(E)#272995; see Table 1 for further details of type specimens examined) and two from Olaa (BMNH(E)#272992 and 272994) arrived as part of accession “[18]99–227”, which according to the NHMUK Accessions Register, was donated by “The Joint Committee of the Royal Society [for Promoting Natural Knowledge] & British Association [for the Advancement of Science]”. These two organizations, together with the Bernice Pauahi Bishop Museum (BPBM, founded 1889) at Honolulu, instituted the explorations under R.C.L. Perkins that led to the production of the “Fauna Hawaiiensis” series, in a volume of which Meyrick [7] described *D*. *pyrias*. The remaining four NHMUK syntypes, all from Olaa (BMNH(E)#272990, 272993, 272996 and one with no specimen registration number), arrived later in 1938 as part of the Edward Meyrick bequest (accession no. B.M. 1938–290).

**Table 1:** Summary of the taxonomy of Hawaiian *Hyles* with a list of type specimens (including exemplars putatively representing type specimens).

Name of specimen	Author	Year	Type	Validity	Valid name	Collector	Date	Locality	Type deposition	Voucher Number	Sex
*Deilephila calida*	Butler	1881	Holotype	invalid	*Hyles calida calida*	Reverend Thomas Blackburn	?	The Hawaiian Islands (“Oahu” according to Rothshild & Jordan, 1903)	NHMUK	BMNH(E) 271654	male
*Deilephila wilsoni*	Rothschild	1894	Holotype	invalid	*Hyles wilsoni*	Scott. B. Wilson	?	Hawaii, Sandwich Islands	NHMUK	None	female
*Deilephila pyrias*	Meyrick	1899	Syntype	invalid	*Hyles wilsoni*	?	11/1896	Hawaii Big Island: Olaa, at 2000 feet	NHMUK	BMNH(E) 272990	male
*Deilephila pyrias*	Meyrick	1899	Syntype	invalid	*Hyles wilsoni*	?	11/1896	Hawaii Big Island: Olaa, at 2000 feet	NHMUK	BMNH(E) 272992	male
*Deilephila pyrias*	Meyrick	1899	Syntype	invalid	*Hyles wilsoni*	?	11/1896	Hawaii Big Island: Olaa, at 2000 feet	NHMUK	BMNH(E) 272996	male
*Deilephila pyrias*	Meyrick	1899	Syntype	invalid	*Hyles wilsoni*	?	11/1896	Hawaii Big Island: Olaa, at 2000 feet	NHMUK	BMNH(E) 272993	female
*Deilephila pyrias*	Meyrick	1899	Syntype	invalid	*Hyles wilsoni*	?	06/1895	Hawaii Big Island: Olaa, at 2000 feet	NHMUK	BMNH(E) 272994	male
*Deilephila pyrias*	Meyrick	1899	Syntype	invalid	*Hyles wilsoni*	?	12/1896	Hawaii Big Island: Olaa, at 2000 feet	NHMUK	None	female
*Deilephila pyrias*	Meyrick	1899	Syntype	invalid	*Hyles wilsoni*	?	01/1896	Hawaii Big Island: Hilo, at 2000 feet	NHMUK	BMNH(E) 272995	male
*Deilephila pyrias*	Meyrick	1899	Syntype	invalid	*Hyles wilsoni*	? (Fauna Hawaiiensis Collection)	01/1896	Hawaii Big Island: Hilo, at 2000 feet	BPBM	None	male
*Deilephila pyrias*	Meyrick	1899	Syntype	invalid	*Hyles wilsoni*	? (Fauna Hawaiiensis Collection)	12/1896	Hawaii Big Island: Olaa[, at 2000 feet]	BPBM	None	male
*Deilephila pyrias*	Meyrick	1894	Syntype	invalid	*Hyles wilsoni*	? (Fauna Hawaiiensis Collection)	[1896]	[Hawaii Big Island: Olaa, at 2000 feet]	BPBM	None	female
*Deilephila pyrias*	Meyrick	1899	Syntype(?)	invalid	*Hyles wilsoni*	? (RCL Perkins collection)	[1896]	Hawaii Big Island: Olaa 1800 ft [Olaa, at 2000 feet]	BPBM	None	female
*Celerio calida hawaiiensis*	Rothshild & Jordan	1915	Holotype	invalid	*Hyles calida hawaiiensis*	H. Palmer	?	Hawaii, Mauna Kea	NHMUK	BMNH(E) 271620	female
*Celerio calida hawaiiensis*	Rothshild & Jordan	1915	Paratype	invalid	*Hyles calida hawaiiensis*	?	?	?	NHMUK	BMNH(E) 271619	male
*Celerio calida hawaiiensis*	Rothshild & Jordan	1915	Paratype	invalid	*Hyles calida hawaiiensis*	R. C. L. Perkins	?	Hawaii, Kau	NHMUK	BMNH(E) 271618	female
*Celerio perkinsi*	Swezey	1920	Lectotype	invalid	*Hyles perkinsi*	J. A. Kusche	bred 11/10/1919, em. 19/11/1919	Hawaiian Islands: Oahu, upper Manoa Valley, Mt. Tantalus	BPBM	None	male
*Celerio perkinsi*	Swezey	1920	Para-lectotype	invalid	*Hyles perkinsi*	Bryan	10/1919	Hawaiian Islands: Oahu, upper Manoa Valley, Mt. Tantalus	BPBM	None	?
*Celerio perkinsi*	Swezey	1920	Para-lectotype	invalid	*Hyles perkinsi*	O. H. Swezey	03/09/1906	Hawaiian Islands: Oahu, Palolo Crater	HDOA	None	male

Inferred information is given in square brackets []. The row listing the specimen sequenced for this study is highlighted in light grey.

In their monumental revision, Rothschild & Jordan [8] transferred both species to the genus *Celerio* Agassiz, 1846 and synonymized *D*. *pyrias* with *C*. *wilsoni*. They gave the type locality of *C*. *calida* as “Oahu”, but it is unclear how they arrived at this conclusion as no specific island was mentioned in the original description, despite such details being provided for many of the other species described by Butler [9], and the specimen label states simply “Hawaiian Islands”. The only specimens of *C*. *calida* that Rothschild & Jordan [8] listed as being in the Tring Museum at that time were “1 ♂ and 1 ♀ (type) in the Tring Museum, the ♀from Mauna Kea”. As implied by Rothschild & Jordan, the ♂ lacks a data label. These two specimens, together with “1 ♀ in the British Museum, bred by R. C. L. Perkins at Kau”, formed the type series (a holotype and two paratypes) of a separate subspecies described over a decade later by Rothschild & Jordan [10] as *Celerio calida hawaiiensis*. The two subspecies were only separated on the basis of the extent of the black basal band of the hindwing upperside (see Kitching [3] for images of all taxa). In *C*. *c*. *calida*, this crosses the whole width of the wing, from the costa to the inner margin, so that the orange coloration is restricted to a discrete, more-or-less parallel-sided medial band. In contrast, in *C*. *c*. *hawaiiensis*, the black basal band is much reduced or absent in the posterior half of the hindwing, and the medial orange coloration extends into much of the basal area. *C*. *c*. *hawaiiensis* had first been illustrated by Rothschild ([11]: pl. IX) (as *Deiphila* [sic] *calida*).

The final Hawaiian taxon to be described was *Celerio perkinsi* Swezey (1920), based on one moth collected while at rest on a tree trunk at Palolo Crater, Oahu; another collected in the upper Manoa Valley on the lower slopes of Mt. Tantalus, Oahu; and a third reared from a small caterpillar given to Swezey by Mr. J.A. Kusche, who found it feeding on leaves of *Straussia*, again on Mt. Tantalus, Oahu. These syntypes were deposited in the collection of the Hawaiian Entomological Society, which is now part of the BPBM.

Shortly before the publication of Rothschild & Jordan’s “*Revision*” on April 21 1903 (see Tutt [12], Tutt ([13] March 15 1903) described the genus *Hawaiina* for the two then known Hawaiian species, *Deilephila calida* (designated as the type species) and *D*. *wilsoni*. The diagnostic characters were given as: “Antennae very long and very stout in ♂; forewings very dark; hindwings deep orange, with dark border reaching to margin”. Given the closeness of the publication dates, it was perhaps inevitable that *Hawaiina* would be overlooked by Rothschild & Jordan [8], but it less clear why the name was not then later noted by either Rothschild & Jordan [10] or Swezey [14]. Eventually, Swezey [15] drew attention to *Hawaiina* (misspelling it as “*Hawaiiana*”) but only explicitly transferred *Deilephila wilsoni*, although he did also mention the other two Hawaiian species (and so the transfer of *Deilephila calida*, the type species, back to *Hawaiina*, was implicit). The remaining two taxa were treated under *Hawaiina* by Swezey [16].

Zimmermann [17] reviewed the Hawaiian *Hyles*, transferring them back to *Celerio* with which he synonymized *Hawaiina* on the basis that the diagnostic characters were not “an adequate reason for erecting a genus for the Hawaiian *Celerio*”. He also synonymized *C*. *perkinsi* with *C*. *wilsoni* as a subspecies but gave no justifiable reason for so doing, stating only “I consider this to be a subspecies of *wilsoni*, instead of a distinct species, in the same way that *hawaiiensis* is considered a subspecies of *calida*”. Zimmermann [17] also implicitly designated the lectotype of *Celerio perkinsi* in the legend to his figure 375. The photograph is sufficient to identify the specimen unequivocally and thus the lectotype designation is valid. Its identity as the specimen bred by J.A. Kusche was determined from photographs sent to us by the BPBM and which we have now made available, with permission, at http://sphingidae.myspecies.info/taxonomy/term/1330/media.

The transfer of all four species to the present genus *Hyles* was made by d’Abrera [18], who also implicitly reinstated *Hyles perkinsi* as a species. However, this status change was not adopted by Nishida [19] in the *Hawaiian Terrestrial Arthropod Checklist*. Kitching & Cadiou [20] provided a justified argumentation as to why *Hyles perkinsi* should be accorded species status on the grounds that the differences between it and *Hyles wilsoni* were much greater than those between *Hyles calida calida* and *Hyles calida hawaiiensis*. Most recently, Danner et al. [21] reinstated *Hawaiina* as one of nine subgenera they recognized/proposed in *Hyles*. However, not only is this rather excessive for a genus of only 30 or so morphologically very close species, but phylogenetic studies using DNA sequence data have repeatedly demonstrated that several of these subgenera are not monophyletic [4, 6] and they should therefore be abandoned as unnecessary.

The phylogenetic position of a Hawaiian *Hyles* within the genus, as sister to the Palearctic species, was first reconstructed using DNA sequence data over 10 years ago [4] although this was based on only the single specimen of *H*. *calida calida* and mitochondrial sequence data only (GenBank accession number AJ749426). Further mitochondrial sequences of *H*. *calida calida* and also of *H*. *perkinsi*, supplemented by nuclear sequence data, allowed Hundsdoerfer et al. [6] to reconstruct a clade of Hawaiian *Hyles* and corroborated its position as sister to the Palearctic *Hyles* (based on total evidence of mitochondrial plus nuclear data). However, it had not been possible to date to include molecular data of the subspecies *H*. *c*. *hawaiiensis* to determine its molecular systematics.

*Hyles calida calida* appears to be quite widespread on the smaller Hawaiian Islands. Zimmermann [17] recorded it from Kauai, Molokai and Oahu, to which we can add records in the NHMUK collection from Lanai and Maui. *Hyles perkinsi* is rather less widely distributed; Zimmerman [17] recorded it from Oahu and Molokai, and there are specimens in NHMUK from Maui. The remaining two taxa are endemic to the “Big Island” of Hawaii, where *H*. *wilsoni* appears to be reasonably frequent (although maybe not as frequent as once believed [22]). However, *Hyles calida hawaiiensis* seems to be very rare. Indeed, Zimmerman [17] gave no details of any additional specimens beyond the types and the only specimens present in the Main Collection of the NHMUK when this study was begun were the three types. Thus, this taxon is something of an enigma. Could it be just a minor colour form of the *Hyles calida* that occur on the other islands and so not worthy of subspecific status? Or might it be more distinct from the remaining *Hyles calida* than the minor diagnostic colour difference suggests? The first aim of this study is to address those questions, because the status of *Hyles calida hawaiiensis*, as synonym, subspecies or even valid species, could have profound implications for the conservation of the taxon. Second, we summarize data on the types of the endemic Hawaiian *Hyles* species to facilitate future taxonomic revision.

## 2. Methods

### 2.1 Material and laboratory techniques

**Fig 1:**
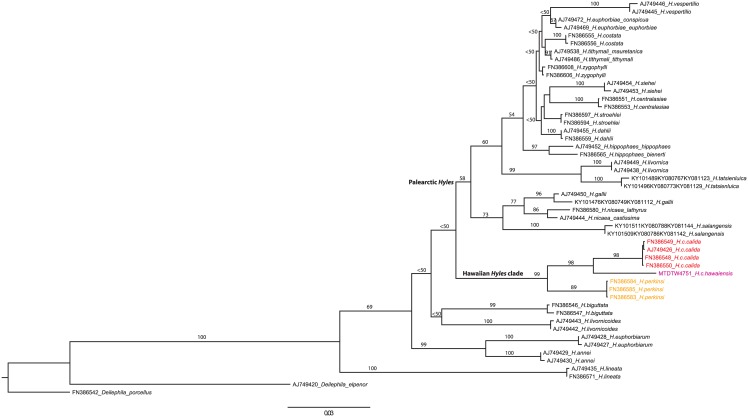
Phylogenetic hypothesis of *Hyles* based on 2,212 bp of mitochondrial sequence data (analysed with RAxML) including the 1,350 bp new sequence data of the ‘historic’ DNA of a century-old museum specimen of *H*. *c*. *hawaiiensis*.

To address the above questions, we sought to sequence ‘historic’ DNA from one of the century-old types of *Hyles calida hawaiiensis*. The abdomen of the paratype reared at Kau by Perkins (BMNH(E) #271618) was macerated following the protocols described by Hundsdoerfer & Kitching [23] (the specimen is illustrated both before and after treatment in their Fig 1A–1D). It is not known exactly when this moth was collected but it came to the then British Museum (Natural History) in 1907 (accession number 1907–347) as part of an exchange. DNA was extracted from the tissue according to the protocol given by Mende & Hundsdoerfer [24]. Due to the expected degradation of the ‘historic’ DNA, the marker genes COI, t-RNA-Leu and COII were amplified in 13 small, overlapping fragments with specifically designed *Hyles* primers following Hundsdoerfer et al. [1]. The sequence was included in a dataset comprising two published sequences of every *Hyles* species available, plus two outgroup sequences from the genus *Deilephila* [1, 4, 6] (GenBank accession numbers are given in the sample labels in Fig 1).

### 2.2 Phylogenetic analyses

To reconstruct the phylogenetic position of *H*. *calida hawaiiensis*, a maximum likelihood (ML) tree with bootstrap support values was reconstructed using RAxML [25] (implemented in raxmlGUI1.5b1 [26]; best of 10 replicate tree searches; GTRCAT; 1000 thorough bootstrap replicates). The best partitioning scheme and evolutionary models were determined with PartitionFinder 1.1.1 [27] using the AICc criterion and the greedy search algorithm (GTR+I+G; three-partition scheme COI1+COII3, COI2+COII1 and COI3+COII2). Distance comparisons were performed with MEGA5 [28].

## 3. Results and discussion

Sequence analysis of the ‘historic’ DNA of the century-plus old museum exemplar of *H*. *c*. *hawaiiensis* yielded a total of 1,350 bp (in several fragments; 39.0% missing data in the 2,212 bp dataset; ‘European Nucleotide Archive’ Accession Numbers LT717725, COI and LT717726, COII). The maximum likelihood phylogram of *Hyles* (Fig 1) corroborates the monophyly of *H*. *calida* and places *H*. *c*. *hawaiiensis* as the sister lineage of a monophyletic *H*. *c*. *calida* (98% bootstrap support), but from which it is well-differentiated (see below). These two taxa together form the sister group of *H*. *perkinsi* (99% bootstrap support) and the Hawaiian clade is, as before, the sister group of the Palaearctic clade (Fig 1). The latter relationship is not supported (bootstrap value < 50%) in this reconstruction, but this is not within the scope of the present paper.

The average p-distance between *Hyles* species in the 2,212 bp dataset (excluding outgroups and *H*. *calida*) is 3.5% (minimum 0.6%, maximum 6.7%); or, alternatively, an average of 74.2 bp differences (minimum 12.5, maximum 132.3). Between the four samples of *H*. *c*. *calida* and the *H*. *c*. *hawaiiensis* there is an average p-distance of 2.8% (or an average of 36.5 bp differences). These values are both well within the range found between other *Hyles* species and are very similar to those between the generally accepted species, *H*. *nicaea* (von Prunner, 1798) or *H*. *salangensis* (Ebert, 1969) and *H*. *costata* (von Nordmann, 1851), *H*. *nicaea* and *H*. *vespertilio* (Esper, 1780), or even between *H*. *livornica* (Esper, 1780) and *H*. *stroehlei* Eitschberger, Danner & Surholt, 1998 (all these comparisons have a p-distance of 2.8%), or *H*. *centralasiae* (Staudinger, 1887) and *H*. *zygophylli* (Ochsenheimer, 1808) (36.75 bp differences). On the basis of these results, *H*. *c*. *hawaiiensis* could be raised to species status. However, although the present result regarding *H*. *c*. *hawaiiensis* is clear, it is nevertheless based on (fragmented) mitochondrial sequences from only a single individual. Further individuals should be analysed using the same marker genes, and also nuclear sequences, as well as a more detailed comparative morphological study, prior to taxonomic revision. Fresh tissue will be necessary to obtain molecular data of nuclear marker genes.

The museum exemplar analysed provides an opening to further systematic research on the entire Hawaiian *Hyles* radiation. The holotypes of *H*. *c*. *calida* and *H*. *wilsoni*, as well as the holotype and two paratypes of *H*. *c*. *hawaiiensis*, are available in the NHMUK collection (see Introduction). At the time that the present study was conceived, these were the only three specimens of this taxon that were known to us. However, there are two additional (female) exemplars in the entomological collection of the Hawaii Department of Agriculture (HDOA) with data: “Hilo, Hawaii, 5-30-1922, Swezey coll.” and “Naalehu, Hawaii 12-7-1905”. There are no moths labelled *H*. *c*. *hawaiiensis* (or *Celerio c*. *hawaiiensis*) in the BPBM. That the subspecies had not apparently been recollected in over a century even led to speculation that it may have become extinct. Finally, during recent recuration of the Hawaiian *Hyles* in the J.-M. Cadiou collection (now in the NHMUK), a sixth specimen of *H*. *c*. *hawaiiensis* was discovered. It, too, is a female, with data: “Hawaiian Is., Hawaii, Kalaoa, 7 mi. N, 2000’, 23.II.1961, D.F. Hardwick”; “Cadiou Coll., BMNH(E) 2008–107”. Not only does this specimen show that reports of its extinction were an exaggeration, but it also holds out hope for more complete sequences being obtainable should no further, more recently collected, specimens be forthcoming. A thorough examination of *H*. *calida* in the BPBM collection should be undertaken, as well as extensive field work on the entire Hawaiian Archipelago, with a focus on Big Island. The youngest known specimen of *H*. *c*. *hawaiiensis* (1961, see above) was collected at Kalaoa, which is thus the best place to start the search.

Not only is the current distribution of *H*. *c*. *hawaiiensis* unknown, but we also lack life history data for the putative species. Despite one of the paratypes being reared by Perkins at Kau, the larval hostplant was not recorded, and remains unknown. Larvae of *H*. *c*. *calida* and *H*. *wilsoni* are both reported to feed on *Acacia koa*, *Bobea*, *Metrosideros*, *Pelea* and *Psychotria* (as the junior synonym, *Straussia*), whereas *Euphorbia*, *Kadua* and *Psychotria kaduana* (as *Straussia kaduana*) are reported for *H*. *perkinsi* [17]. *Euphorbia* is listed additionally for *H*. *wilsoni*, and *Coprosoma*, *Gardenia* and *Scaevola* for *H*. *c*. *calida*. As all the other three endemic Hawaiian *Hyles* taxa use *Psychotria*, the larvae of *H*. *c*. *hawaiiensis* would also be expected to feed on this plant genus, or other related Rubiaceae, such as *Coprosoma* or *Gardenia*. *Euphorbia* (Euphorbiacae) is only listed for the sister-pair *H*. *wilsoni* and *H*. *perkinsi* and not for *H*. *c*. *calida*, so *H*. *c*. *hawaiiensis* larvae would probably not be expected to utilize this plant.

Our understanding of morphological characters in *Hyles* and their utility in resolving the phylogenetic relationships of the species has changed markedly since Hundsdoerfer et al. [4] revealed that similar morphology does not necessarily imply monophyly or even a ‘close’ phylogenetic relationship. Thus, for example, the note of Swezey ([14]: 380) would now be considered erroneous: “NOTE.—Recently the Bishop Museum has received, by way of exchange, a pair of *Celerio galli* [sic] *intermedia* Kirby from Mr. B. Preston Clark of Boston. These specimens were collected in Alaska. The species is widely distributed in North America. Comparing these with the Hawaiian *Celerios*, there is a striking similarity in the color patterns of the wings, and the abdominal markings. The similarity is most striking with our *C*. *calida* (Butler). It seems to me to indicate the American origin of the ancestors of the Hawaiian *Celerios*. Mr. Clark, in letter, also concurs in the belief that the Hawaiian Sphingidae are allied to those of America.” It has now been shown by Hundsdoerfer et al. [1] that North American *Hyles gallii* (von Rottemburg, 1775) show no molecular sequence differentiation from Palaearctic *H*. *gallii* and certainly do not form a clade with the Hawaiian *Hyles* (an Alaskan *H*. *gallii* specimen was included in Fig 1, GenBank accession numbers KY101476, KY080749, KY081112); nor, for that matter, do the other “American” species, *H*. *annei* (Guerin-Méneville, 1839), *H*. *euphorbiarum* (Guérin-Méneville & Percheron, 1835) and *H*. *lineata* (Fabricius, 1775).

Hundsdoerfer et al. [1] reconstructed the ancestral ranges of all available *Hyles* species. The postulated sister group relationship between the Palaearctic and Hawaiian *Hyles* (Fig 1; [4, 6]) was corroborated and the split was dated to 2.95–5.12 Ma. Colonisation of Hawaii by *Hyles* has been postulated to have possibly taken place via the Bering Strait [4, 6]. Sea ice-related diatom species appeared in the Bering Strait at 2.78–2.55 Ma [29], marking the regional cooling associated with the expansion of Northern Hemisphere ice sheets and corresponding well with the youngest date estimate of the origin of the Hawaiian clade. Glaciation in the area of the Bering Strait may have forced the strong flying hawkmoths southwards to search for warmer habitats, reaching the Hawaiian archipelago after crossing stretches of ocean [4]. Any potential role for the Northwestern Hawaiian Islands must be purely speculative. These now small islands and atolls, such as Nihoa, Necker and Midway, would have been above water at the appropriate time but we have found no references to the presence of *Hyles* there now or in the past. The oldest rock in the present day main Hawaiian islands, that of Kaua‘i, is about 5.5 Ma old [30] and deeply eroded. At that time, the sea level was only 25 m higher than today [31] and thus that island might be expected to have had potential habitat in the Pliocene for the ancestors of today’s Hawaiian *Hyles*.

The assembly of data available on the type material of Hawaiian *Hyles* (Table 1) led to two discoveries: 1. Three of the missing syntypes of the original description of *Deilephila pyrias* have been located in the collection of the Hawaii State Museum of Natural and Cultural History (BPBM) (Table 1). They should receive syntype lables. One is labelled “Hilo, Hawaii, 2000ft, 1.96” “Fauna Hawaiiensis Collection” “Nakamura 2002 pollen slide 757” (male); a second is labelled “Olaa, xii.96” “Fauna Hawaiiensis Collection” (male); and the third is labelled only “Fauna Hawaiiensis Collection” (female; Table 1). However, this last specimen will be from Olaa as both Hilo specimens have been accounted for. A fourth specimen in the BPBM is labelled “Olaa 1800ft” “RCL Perkins Collection” (female). Although it lacks the “Fauna Hawaiiensis Collection” label and the altitude is different from that given in the original description, it is most probably the final syntype as “a considerable number of specimens [were] given to Dr Perkins for his assistance in Honolulu” (Sharp [32]).

2. The three specimens of *Celerio perkinsi* on which the species description was based were originally deposited in the Hawaiian Entomological Society collection, which is now part of the BPBM, and this was where we had thought that all three specimens were deposited today. However, while the lectotype and one of the two paralectotypes of *Celerio perkinsi* are indeed in the BPBM, we discovered the second paralectotype is now in the HDOA entomological collection.

## 4. Conclusions

The degree of divergence between the ‘historic’ DNA of the 100+ year-old specimen of *H*. *c*. *hawaiiensis* and several samples of *H*. *c*. *calida* suggests that the former taxon should perhaps be awarded species status. However, more data are needed for a sound taxonomic revision. The reduced or nearly absent black basal band of the hindwing upperside is certainly diagnostic for *H*. *c*. *hawaiiensis*, but its adaptive significance remains obscure. While wing patterns allow easy field determination of specimens, rigorous analyses of an explicitly coded matrix of morphological characters mapped onto a phylogenetic tree derived from comprehensive molecular data would be the minimum necessary for a better understanding of wing pattern evolution in the genus, for which freshly collected individuals of both taxa would be required. Given the rarity of this taxon, these analyses should be undertaken urgently so that conservation measures can be implemented before it becomes extinct.
